# Targeting Glutathione *S*-transferase M4 in Ewing sarcoma

**DOI:** 10.3389/fped.2014.00083

**Published:** 2014-08-06

**Authors:** Rupeng Zhuo, Kenneth M. Kosak, Savita Sankar, Elizabeth T. Wiles, Ying Sun, Jianxing Zhang, Janet Ayello, Glenn D. Prestwich, Paul J. Shami, Mitchell S. Cairo, Stephen L. Lessnick, Wen Luo

**Affiliations:** ^1^Center for Children’s Cancer Research, Huntsman Cancer Institute, University of Utah, Salt Lake City, UT, USA; ^2^Department of Internal Medicine, Division of Hematology and Hematologic Malignancies, University of Utah, Salt Lake City, UT, USA; ^3^Microarray and Genomic Analysis Core Facility, Huntsman Cancer Institute, University of Utah, Salt Lake City, UT, USA; ^4^Department of Medicinal Chemistry, University of Utah, Salt Lake City, UT, USA; ^5^Department of Pediatrics, New York Medical College, Valhalla, NY, USA; ^6^Department of Medicine, New York Medical College, Valhalla, NY, USA; ^7^Department of Microbiology and Immunology, New York Medical College, Valhalla, NY, USA; ^8^Department of Cell Biology and Anatomy, New York Medical College, Valhalla, NY, USA; ^9^Department of Pathology, New York Medical College, Valhalla, NY, USA; ^10^Division of Pediatric Hematology/Oncology, School of Medicine, University of Utah, Salt Lake City, UT, USA

**Keywords:** GSTM4, Ewing sarcoma, NBDHEX, JS-K, drug resistance

## Abstract

Ewing sarcoma is a malignant pediatric bone and soft tissue tumor. Although the 5-year survival rate of localized disease approaches 75%, the prognosis of metastatic and/or therapy-resistant disease remains dismal despite the wide use of aggressive therapeutic strategies. We previously reported that high expression of glutathione *S*-transferase M4 (GSTM4) in primary tumors correlates with poor patient outcomes. GSTM4 is required for oncogenic transformation and mediates resistance to chemotherapeutic drugs in Ewing sarcoma cells. Here, we performed RNA-sequencing analyses of Ewing sarcoma cells and combined our results with publicly available datasets to demonstrate that GSTM4 is a major GST specifically expressed in Ewing sarcoma. Pharmacological inhibition of GSTM4 activity using a pan GST inhibitor, 6-(7-nitro-2,1,3-benzoxadiazol-4-ylthio) hexanol (NBDHEX), significantly limited cellular proliferation and oncogenic transformation of Ewing sarcoma cells. Moreover, combined use of NBDHEX and etoposide synergistically increased cytotoxicity, suggesting a role for GSTM4 as an inhibitor of apoptosis. Mechanistic studies revealed that GSTM4 limits apoptosis owing to its ability to interact with Apoptosis Signal-regulating Kinase 1 (ASK1) and inhibit signaling via the c-Jun N-terminal Kinase axis. To exploit our observation that GSTM4 expression is specifically up-regulated in Ewing sarcoma, we tested the effect of a GSTM4-activated anti-cancer agent, O^2^-(2,4-dinitrophenyl) 1-[(4-ethoxycarbonyl)piperazin-1-yl]diazen-1-ium-1,2-diolate or JS-K, on tumor growth and survival. We found that JS-K robustly decreased Ewing sarcoma cell viability and xenograft tumor growth and improved overall survival of xenograft mice. Our data suggest that GSTM4 is a novel therapeutic target for the treatment of high GSTM4-expressing Ewing sarcoma. Strategies that combine standard chemotherapy with agents that inhibit GSTM4, that are activated by GSTM4, or that block GSTM4/ASK1 interactions, can potentially be more specific and/or efficacious than standard therapeutic approaches.

## Introduction

Glutathione *S*-transferases (GSTs) comprise a family of detoxification enzymes that catalyze the conjugation of glutathione with carcinogens, drugs, toxins, and products of oxidative stress (1, 2). It is believed that the function of these enzymes is to reduce the incidence of deleterious interactions between reactive toxic species and cellular components. GSTs are highly polymorphic and can be divided into eight subfamilies, α (GSTAs), κ (GSTKs), μ (GSTMs), ω (GSTOs), π (GSTPs), θ (GSTTs), ζ (GSTZs), and membrane-bound GSTs (MGSTs). Each subfamily is composed of several members with similarities in either primary sequence or structure (3). Although GSTs may utilize “common” substrates such as CDNB (1-chloro-2,4-dinitrobenzene) *in vitro*, individual GSTs have unique substrate specificities, suggesting that each family member plays a defined role in biotransformation of drugs or reactive compounds.

Glutathione *S*-transferases have been implicated in the development of resistance to cancer chemotherapeutic agents (4). High levels of GST expression have been reported in a number of tumors compared to normal tissues. Overexpression of MGST1 and GSTP1 was found to induce doxorubicin and cisplatin resistance in MCF-7 breast cancer cells (5), pointing at their potential functional roles in chemoresistance. Consistently, high MGST1 expression in Ewing sarcoma correlates with poor prognosis due to doxorubicin resistance (6). It seems that GSTs play two distinct roles in the development of drug resistance. First, the enzymes can directly inactivate anti-cancer agents that act as substrates by catalyzing their conjugation to GSH. Second, GSTs can inhibit the MAP kinase pathway and limit apoptosis induced by drugs that are not GST substrates (7–9). Because of their overexpression in a wide variety of cancers and their key role in drug resistance, GST inhibition has emerged as a promising therapeutic approach to limit resistance to cancer therapeutics.

Ewing sarcoma is the second most common pediatric bone and soft tissue tumor. Current treatment for this malignancy consists of local control via surgery and/or radiation, and intensive chemotherapy. For patients with localized disease, expected survival rates are reaching 75% with these aggressive therapies. However, the outcome for patients with metastatic tumors remains poor, with a 5-year survival <30%. Novel treatment strategies targeting specific regulators of Ewing sarcoma are urgently needed. Most cases of Ewing sarcoma are driven by the fusion oncoprotein EWS/FLI that results from a chromosomal translocation between chromosomes 11 and 22. EWS/FLI is an aberrant transcription factor that deregulates expression of target genes, leading to the tumorigenic and undifferentiated phenotypes of Ewing sarcoma (10). We previously reported that GSTM4, a relatively new member of the μ class of GSTs, is a direct, up-regulated, target of EWS/FLI. Patients who express high GSTM4 levels in their primary tumors have worse outcomes compared with those whose tumors express lower levels of GSTM4 (11). In addition, GSTM4 actively contributes to tumorigenesis and drug resistance in Ewing sarcoma. Knockdown of GSTM4 decreases oncogenic transformation and increases the sensitivity of Ewing sarcoma cells to chemotherapeutic drugs. These combined observations suggest that GSTM4 is a promising novel target to treat Ewing sarcoma.

## Materials and Methods

### Cell lines and reagents

Ewing sarcoma cell lines A673 and TC71 were purchased from American Type Culture Collection (ATCC, Manassas, Virginia) and were cultured as previously described (12). Growth curve and soft agar colony formation assays were performed as before (12). Etoposide was purchased from Sigma (St. Louis, MO, USA). NBDHEX was synthesized as previously described (13). Briefly, NBD chloride (1 mmol) and 6-mercapto-1-hexanol (2 mmol) were dissolved in 20 mL of a 1:1 (v/v) mixture of ethanol and 0.1 M potassium phosphate buffer (pH 7.0) at room temperature. The resulting mixture was stirred for 6 h. The product, NBDHEX, was collected by filtration and washed twice with distilled water and dried *in vacuo*. JS-K was synthesized as previously described (14).

### Constructs

The construct pcDNA3–ASK1 was generously provided by Dr. Sheng-Cai Lin (Xiamen University, China). GSTM4 full length construct and deletion mutants GSTM4-NT and GSTM4-CT were generated by amplifying the full length, 1–222 and 223–656 bp of *GSTM4*, respectively, by PCR and cloning the fragments into the pCS2-MT vector. RNAi constructs for GSTM4 (GSTM4-4-RNAi and GSTM4-5-RNAi) and control (Luc-RNAi) were generated and used as previously described (11).

### MTT cell proliferation assay

A673 or TC71 cells were seeded in a 24-well plate at a density of 5 × 10^4^ cells/well and then incubated at 37°C for 24 h. Following drug addition, the cells were cultured for 72 h. Each condition was performed in triplicate. MTT assays were carried out using a reagent provided by Cayman Chemical (10009365, Ann Arbor, MI, USA), and following recommendations provided by the manufacturer. Bliss combination index (CI) values were calculated to assess synergistic (CI <1), additive (CI = 1), or antagonistic (CI >1) effects of combined drugs on cytotoxicity as previously described (15).

### RNA-seq and data analysis

Total RNA was extracted from Ewing sarcoma cells and treated with DNase using a Qiagen (Germantown, MD, USA) RNeasy kit. Messenger RNA was enriched by oligo-dT magnetic beads and was used to construct Illumina sequencing libraries. The libraries were single end-sequenced on Illumina Genome Analyzer IIx for 36 cycles. Reads were mapped to the hg19 genome build with Casava. The RNA-seq analysis was carried out using USeq (useq.sourceforge.net) version 8.1.5. Sorted mapped files were converted to PointData representation with the USeq Eland Parser application. The raw data can be accessed from the Sequence Read Archive (SRA) database (accession number SAMN01163407).

### Immunoprecipitation and immunoblotting

Immunoprecipitation and immunoblotting were carried out as previously described (16). Anti-ASK1 (sc-5294 and sc-390275) and anti-Myc (sc-40) antibodies were from Santa Cruz Biotechnology (Dallas, TX, USA). Anti-Jun N-terminal kinase (JNK) (9252) was from Cell Signaling Technology (Beverly, MA, USA).

### Kinase assay

Jun N-terminal kinase kinase assays were carried out using a non-radioactive MAPK/JNK kinase assay kit from Cell Signaling Technology (Beverly, MA, USA), following the manufacturer’s instructions.

### Animal engraftment and treatment

To test the efficacy of combined etoposide and NBDHEX treatment, TC71 cells (1 × 10^6^) were injected with matrigel into the flanks of 4–6-week-old female nude mice. Mice were randomly grouped into four groups 24 h after injection. Mice in the control group were treated with vehicle [0.5% Methocel (w/v)] only; animals in the “etoposide” group received a single dose of etoposide (18 mg/kg, i.p.); the “NBDHEX” group consisted of mice receiving 40 mg/kg of NBDHEX daily for 7 days by gavage; and the “combination” group included treatments with both etoposide and NBDHEX. To test the efficacy of JS-K, TC71 cells (1 × 10^6^) were injected with matrigel into both flanks of 4–6-week-old nude mice, followed by vehicle (2.25% Pluronic^®^ P123 in PBS/2% DMSO) or JS-K treatment (6 μmol/kg) intravenously starting 24 h later and continuing every other day for 2 weeks. Tumor growth was monitored by caliper measurement; three-dimensional tumor volumes were calculated using the equation (Length × Width × Depth)/2. The *p*-value for tumor growth curve was determined by the ANOVA test using GraphPad Prism. The mice were sacrificed once a tumor reached a size of 2 cm. Percent survival was plotted as Kaplan–Meier survival curves using GraphPad Prism. The *p*-value was determined by the log-rank (Mantel–Cox Test) method using GraphPad Prism. Animal experiments were performed following approval from the University of Utah Institutional Animal Care and Use Committee.

## Results

### GSTM4 is one of the major GSTs expressed in Ewing Sarcoma

**Figure 1:**
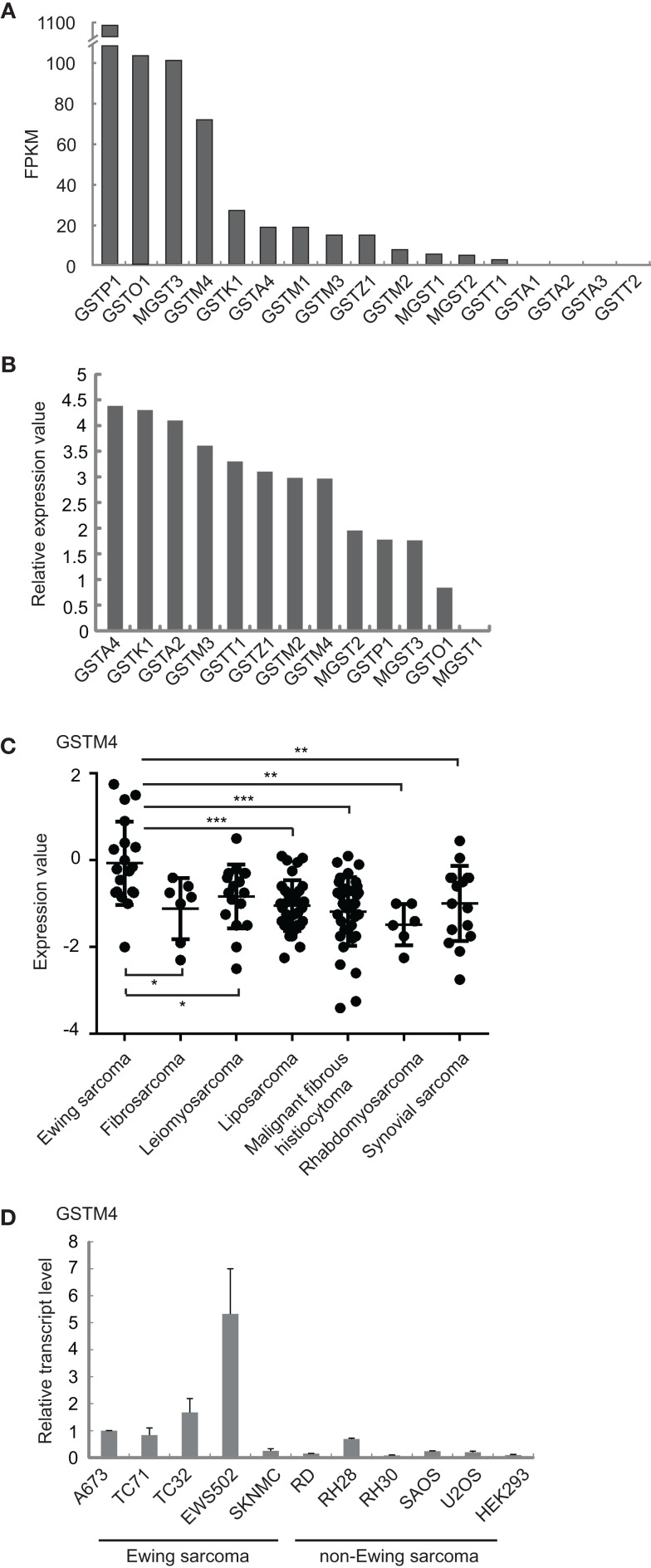
**GSTM4 is a major GST specifically expressed in Ewing sarcoma**. **(A)** Expression levels of all detectable GSTs in A673 cells. Total RNA extracted from A673 cells was subjected to cDNA library construction and labeling, followed by next generation sequencing using Illumina HiSeq1000, as described previously (17). GST genes are ranked by value of FPKM (Fragments per kilobase per million mapped reads). **(B)** Relative expression levels of GSTs in tumors from Ewing sarcoma patients. Expression values of GSTs from the Baird et al. dataset (18) were normalized to that of MGST1, which was set as 0. **(C)** GSTM4 expression levels in tumors from patients with various sarcomas. Data were extracted from the Baird et al. dataset. Horizontal lines indicate mean values. ANOVA statistical analysis results are shown (*P < 0.05; **P < 0.01; ***P < 0.001). **(D)** GSTM4 expression levels in various sarcoma cell lines. Total RNA was extracted from Ewing sarcoma (A673, TC71, TC32, EWS502, SKNMC), rhabdomyosarcoma (RD, RH28, RH30), osteosarcoma (SAOS, U2OS), and HEK293 cells followed by quantitative RT-PCR analyses. The relative GSTM4 transcript levels normalized to GAPDH were shown. The experiment was repeated three times and results of one representative repeat are shown as means ± SD of technical triplicate.

We previously found that GSTM4 is an up-regulated target of the Ewing sarcoma oncoprotein EWS/FLI (11). To determine whether this feature is unique to GSTM4 or common to other GST members, we characterized the expression profile of all known GST family members in patient-derived A673 Ewing sarcoma cells using RNA-seq (17). We found that GSTM4 is expressed at high levels and ranks fourth among 17 members of the family (Figure 1A). In addition to GSTM4, GSTP1, GSTO1, and MGST3 are prevalent GSTs in A673 cells. We next asked whether GSTM4 is also highly expressed in Ewing sarcoma from human patients. Using a publicly available gene expression profile dataset of 19 Ewing sarcoma tumors (18), we again found that GSTM4 was one of the major GSTs expressed in Ewing sarcoma tumors (Figure 1B). We previously reported that EWS/FLI regulates GSTM4 expression via a GGAA microsatellite in the promoter region of the *GSTM4* gene (11). Notably, this GGAA microsatellite regulatory element is not present in any other *GST* promoters, suggesting that GSTM4 may be specifically expressed in Ewing sarcoma due to expression of the EWS/FLI oncoprotein in these malignancies. To investigate this issue, we compared GSTM4 expression levels in various sarcoma tumors using the Baird et al. data set (18), and in cell lines using qRT-PCR. We found very low GSTM4 expression in most non-Ewing sarcoma tumors and cell lines (Figures 1C,D). This analysis also revealed that high GSTM4 expression was typical of approximately half of the Ewing sarcoma tumors included in the Baird et al. dataset. This suggests that factors additional to EWS/FLI likely participate in the regulation of GSTM4 expression. We attempted to compare GSTM4 protein level in various sarcoma cell lines and tumors. However, we could not find a GSTM4-specific antibody that is capable of detecting GSTM4 at endogenous level. In conclusion, GSTM4 transcripts constitute one of the major GST transcripts specifically expressed in Ewing sarcoma cells and tumors.

### Inhibition of GSTM4 by NBDHEX decreases cell viability and inhibits oncogenic transformation of Ewing Sarcoma cells

**Figure 2:**
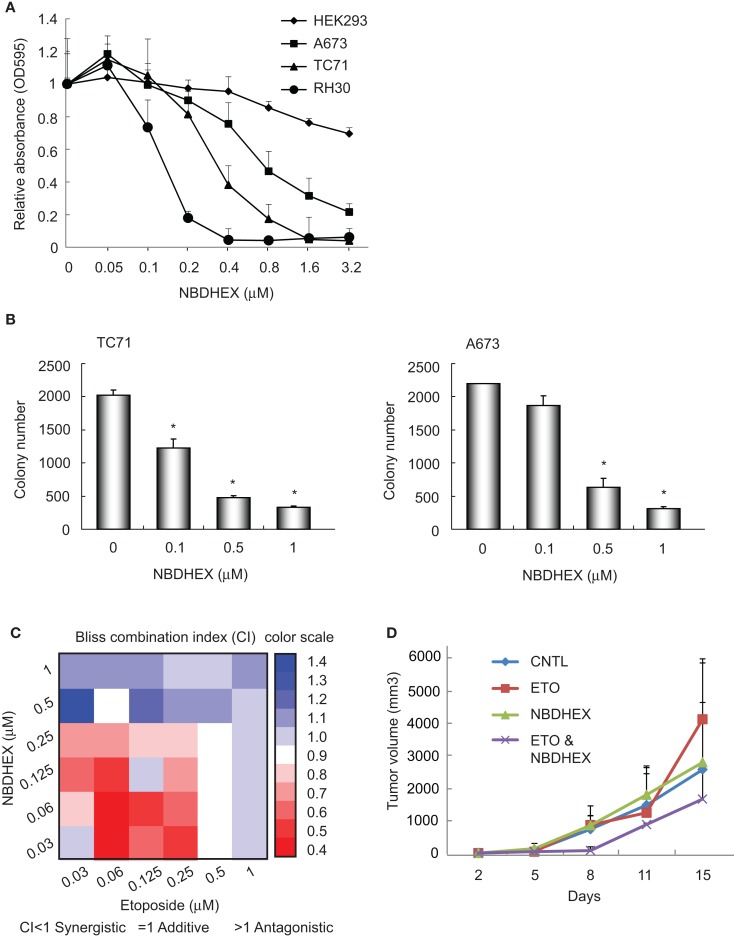
**The GST inhibitor NBDHEX inhibits Ewing sarcoma cell proliferation and oncogenic transformation and increases the efficacy of etoposide**. **(A)**, NBDHEX inhibits Ewing sarcoma cell growth in culture. Ewing sarcoma TC71 and A673 cells, rhabdomyosarcoma RH30 cells, and HEK293 cells were subjected to treatment with the indicated concentrations of NBDHEX. Cell proliferation was assessed by MTT assays. The experiment was repeated three times and results of one representative repeat are shown as means ± SD of technical triplicate. **(B)** NBDHEX inhibits colony formation of Ewing sarcoma cells in soft agar. TC71 and A673 cells were treated with NBDHEX at the indicated concentrations, and then were seeded in soft agar to assess anchorage-independent growth. The experiment was repeated three times and results of one representative repeat are shown as means ± SD of technical duplicate. Asterisk indicates *p* < 0.05. **(C)** NBDHEX and etoposide act synergistically in inducing cytotoxicity of Ewing sarcoma cells. Cells were treated with NBDHEX, etoposide, or NBDHEX together with etoposide, as indicated. Cellular proliferation was then assessed by MTT assays, and bliss combination index (CI) was calculated and represented as a heatmap. CI <1 indicates synergism, =1 additive effect, and >1 antagonism. **(D)** Effect of combined NBDHEX and etoposide treatment on Ewing sarcoma xenograft tumors. TC71 cells were injected subcutaneously into both flanks of nude mice. NBDHEX, etoposide, or NBDHEX plus etoposide were administered 24 h after cell injection. Tumor growth was monitored by caliper measurement twice a week. Each group contained 5 animals with total of 10 tumors. The experiment was repeated three times and results of one representative repeat are shown as average of 10 tumors ± SD. ANOVA test indicates the differences in tumor growth between combination group and the other three groups are not statistically significant (p = 0.123).

Our previous observation that GSTM4 is required for oncogenic transformation and mediates etoposide resistance of Ewing sarcoma cells, led us to hypothesize that GSTM4 inhibitory agents might be cytotoxic and increase the sensitivity of Ewing sarcoma cells to etoposide. To test this, we treated A673 and TC71 cells with 6-(7-nitro-2,1,3-benzoxadiazol-4-ylthio) hexanol (NBDHEX), an effective GST inhibitor and new anti-cancer agent. HEK293 and RH30 cells were used as negative and positive controls, respectively (19). We found drastic decrease in cell proliferation in RH30, A673, and TC71, but not HEK293 cells in response to NBDHEX treatment (Figure 2A). This is consistent with a previous report showing that Ewing sarcoma cell lines are sensitive to NBDHEX (6). Moreover, when we seeded NBDHEX-treated cells in soft agar, these cells formed significantly fewer colonies in the anchorage-independent environment, even when treated with NBDHEX at concentrations much lower than its reported IC_50_ [1 μM (19), Figure 2B]. These results indicate that inhibition of GSTM4 decreases cellular proliferation and abolishes oncogenic transformation. The data are in agreement with our previous finding that GSTM4 knockdown inhibits oncogenic transformation (11).

### NBDHEX and etoposide have synergistic effects on cytotoxicity in Ewing Sarcoma cells

We previously found that knockdown of GSTM4 renders Ewing sarcoma cells more sensitive to etoposide (11). To evaluate whether GSTM4 inhibition has similar effects, we treated A673 and TC71 cells with NBDHEX, etoposide, or NBDHEX combined with etoposide. Bliss CI values then were calculated (15) to assess whether NBDHEX and etoposide have synergistic, additive, or antagonistic effects on cytotoxicity (CI <1 indicates synergism). We found that etoposide is more effective when combined with NBDHEX than either etoposide or NBDHEX alone, indicating a synergistic effect (Figure 2C). Interestingly, synergism between the agents was much stronger at low drug levels (Figure 2C). We next tested if combined NBDHEX and etoposide treatment affected xenograft tumor growth *in vivo*. We found robust synergism during early phases of tumor development (8 days, Figure 2D). This effect was less pronounced at later times, as tumors developing in the combination group continued to grow and reached sizes only slightly smaller than tumors developing in the single treatment groups (Figure 2D, ANOVA test *p* = 0.123). Lack of significant synergism at later time points may be due to incomplete inhibition of GSTM4 by NBDHEX despite daily dosing for 7 days. NBDHEX forms a covalent sigma complex with glutathione that binds tightly to GSTM4 (13). Thus, while binding of the inhibitor to the enzyme–substrate complex is tight, it is reversible, and the slow-tight binding inhibition may lead a plateau of extended incomplete inhibition with continuous dosing. We observed no significant difference in overall survival among the treatment groups. Additionally, the most significant and largest inhibition of tumor growth was observed when both drugs were injected at very low doses, that is, at levels where single agents had minimal or no effects on tumor growth (data not shown). These results are consistent with our findings in cellular models and they suggest that combining NBDHEX and etoposide at low drug levels synergistically increases cytotoxicity, thus minimizing potential high dose-related side effects.

### GSTM4 interacts with ASK1 and inhibits etoposide-induced JNK activation and apoptosis

We next investigated the mechanism of GSTM4-mediated resistance to etoposide. We previously found that depletion of GSTM4 caused no significant alterations in reactive oxygen species production (11), indicating that the ability of GSTM4 to increase drug resistance is independent of its transferase activity. In addition to detoxifying electrophiles and toxic agents by catalyzing their conjugation to reduced GSH, GSTs can bind to various proteins and serve as regulators of MAP kinase-mediated apoptosis. Mouse GSTM1 was found to physically interact with Apoptosis Signaling Kinase 1 (ASK1), leading to repression of ASK1-mediated stress-activated signals and apoptosis (9). Similarly, GSTA1 and GSTP1 have been shown to interact with JNK and inhibit apoptosis (7, 8).

**Figure 3:**
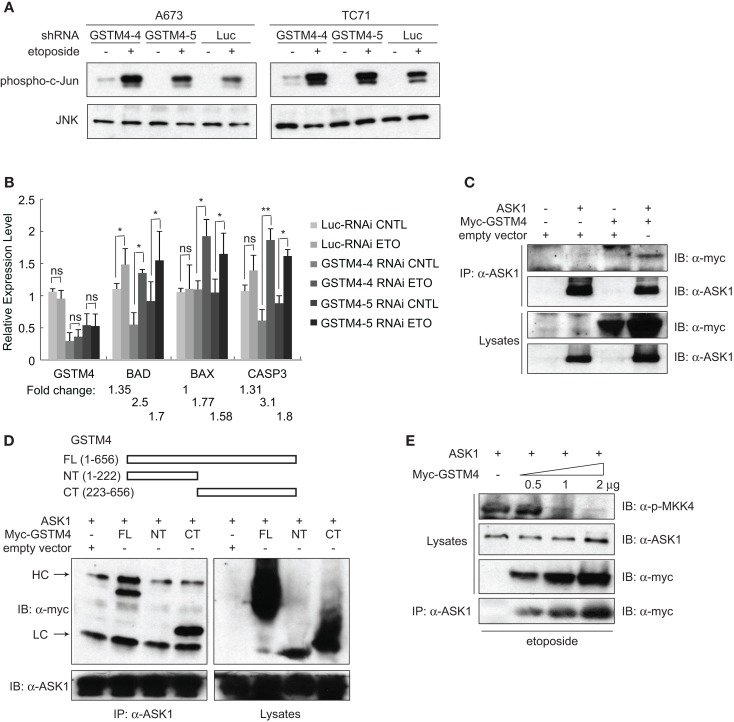
**GSTM4 inhibits etoposide-mediated JNK activation and apoptosis by interacting with ASK1**. **(A)** Decreasing GSTM4 levels increases JNK activation induced by etoposide. Control- (Luc-RNAi) and GSTM4-silenced (GSTM4-4-RNAi and GSTM4-5 RNAi) cells were treated with or without etoposide (20 μg/ml) for 3 h. Cell lysates were subjected to JNK immunoprecipitation and JNK kinase assays using c-jun as the substrate. The result shown is representative of three experimental repeats. **(B)** Decreasing GSTM4 levels increases apoptosis induced by etoposide. Control-silenced (Luc-RNAi) and GSTM4-silenced (GSTM4-4-RNAi and GSTM4-5 RNAi) cells were treated with or without etoposide, as in **(A)** above. Expression levels of GSTM4, BAD, BAX, and CASP3 then were evaluated by qRT-PCR analyses. “ns” indicates not significant, *p < 0.05, **p < 0.01. Fold-change in expression levels before and after etoposide treatment are indicated. The result shown is representative of three experimental repeats. **(C)**, GSTM4 interacts with ASK1. Cells were transfected with ASK1, myc-GSTM4, or ASK1 together with myc-GSTM4. Cell lysates were subjected to immunoprecipitation using an anti-ASK1 antibody and the amount of co-immunoprecipitated GSTM4 was assessed using an anti-myc antibody. The result shown is representative of three experimental repeats. **(D)**, ASK1 binds to the C-terminal domain of GSTM4. Cells were transfected with ASK1 and/or GSTM4 deletion mutants, and processed as indicated in **(C)** above. HC, heavy IgG chain; LC, light IgG chain. The result shown is representative of three experimental repeats. **(E)**, Binding of GSTM4 to ASK1 inhibits MKK4 phosphorylation. Cells were transfected with ASK1 and increasing amounts of GSTM4, as indicated. MKK4 phosphorylation was analyzed by immunoblot analysis, using an anti-phosphorylated MKK4 (Thr261) antibody. The result shown is representative of three experimental repeats.

To investigate whether GSTM4 regulates JNK activation and apoptosis, we treated control or GSTM4-silenced A673 and TC71 cells with etoposide and then determined the extent of c-Jun phosphorylation as a measure of JNK activation. We found that etoposide treatment led to higher JNK activation in GSTM4 knockdown (GSTM4-4 and GSTM4-5 RNAi) cells compared with control (Luc-RNAi) cells (Figure 3A), indicating that GSTM4 inhibits JNK activation induced by etoposide. We next characterized expression of the pro-apoptotic genes *BAD*, *BAX*, and *CASP3* in etoposide-treated GSTM4- or control-silenced cells. We found that etoposide had modest or no effect on the expression of these pro-apoptotic genes in control-knockdown cells (Figure 3B). In contrast, expression of the three tested genes was robustly increased in GSTM4-knockdown cells (Figure 3B). These combined data strongly suggest that GSTM4 plays a central inhibitory role in etoposide-induced JNK activation and apoptosis.

To investigate the mechanism by which GSTM4 inhibits apoptosis, we assessed whether GSTM4 interacts with pro-apoptotic kinases in a manner similar to that of other GSTs. We focused our attention on a panel of kinases in the MAPK pathway including JNK, p38, MKK4, MKK7, ASK1, and MEKK1. Immunoprecipitation analyses showed that ASK1, but not other kinases tested (not shown), robustly binds to GSTM4 (Figure 3C). To identify the key GSTM4 region responsible for interaction with ASK1, we generated GSTM4 deletion mutants, GSTM4-NT and GSTM4-CT, in which the substrate binding site and GSH binding site of GSTM4 are absent, respectively (Figure 3D, upper panel). Immunoprecipitation studies using these deletion mutants showed that the C-terminal end of GSTM4 is required for interaction with ASK1 (Figure 3D, lower panel). However, further studies are necessary to determine whether this interaction is direct.

To study the mechanism whereby GSTM4/ASK1 complexes inhibit etoposide-induced JNK activation and apoptosis, we over-expressed ASK1 and GSTM4 at various ratios, evaluated the extent of GSTM4/ASK1 complex formation, and then assessed the extent of phosphorylation of MKK4, the MAP2K that functions downstream of ASK1, and that is required for JNK phosphorylation and activation. We found that as interaction between GSTM4 and ASK1 increased, the extent of MKK4 phosphorylation decreased (Figure 3E). Taken together, our data suggest that GSTM4 binds to ASK1 and inhibits JNK activation and apoptosis induced by etoposide, thus increasing resistance of Ewing sarcoma cells to this commonly used cancer therapeutic agent.

### The GST-activated pro-drug JS-K inhibits cellular proliferation and xenograft tumor growth via GSTM4

**Figure 4:**
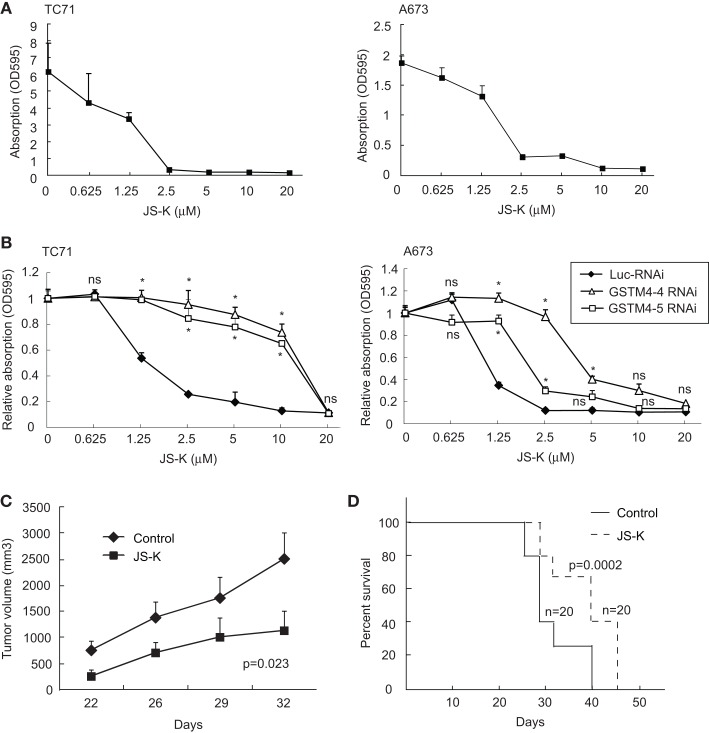
**JS-K is a GSTM4-activated drug that abolishes cell proliferation and xenograft tumor growth**. **(A)** JS-K is effective in inhibiting TC71 and A673 Ewing sarcoma cell growth. TC71 (left) and A673 (right) cells were seeded in 24-well plates, allowed to grow to confluence, and then treated with the indicated concentrations of JS-K for 72 h. MTT assays then were performed to assess cellular proliferation. The experiment was repeated three times and results of one representative repeat are shown as means ± SD of technical duplicate. **(B)** GSTM4-silenced cells are more resistant to JS-K. Control-silenced (Luc-RNAi) or GSTM4-silenced (GSTM4-4-RNAi and GSTM4-5 RNAi) TC71 (left) or A673 (right) cells were subjected to JS-K treatment, as indicated. Cellular proliferation was assessed by MTT assays. The experiment was repeated three times and results of one representative repeat are shown as means ± SD of technical duplicate. Asterisk indicates that the difference between control-silenced and GSTM4-silenced cells is significant (*p* < 0.05); ns, not significant. **(C)** JS-K inhibits Ewing sarcoma xenograft tumor growth in immunodeficient mice. TC71 cells were injected subcutaneously into both flanks of nude mice. JS-K was administered via tail vein injection the day after cell injection every other day for 2 weeks. Tumor growth was monitored by caliper measurement twice a week. The experiment was repeated four times and results of one representative repeat are shown as means ± SD of 10 tumors in each group. ANOVA test, *p* = 0.023. **(D)** Overall survival of vehicle or JS-K-treated mice harboring tumor xenografts. Kaplan–Meier survival curve was plotted using GraphPad Prism. Mantel–Cox Test, *p* = 0.0002.

Since GSTM4 is a major GST specifically expressed in EWS/FLI-driven Ewing sarcoma cells and tumors, drugs preferentially activated by GSTM4 (i.e., GSTM4 pro-drugs) may be attractive therapeutics to target drug-resistant Ewing sarcoma. O^2^-(2,4-dinitrophenyl) 1-[(4-ethoxycarbonyl)piperazin-1-yl] diazen-1-ium-1,2-diolate or JS-K is a GST-activated pro-drug and a member of the O^2^-aryl diazeniumdiolate compound family. Activation of JS-K by GSTs releases nitric oxide (NO) and induces apoptosis (20). JS-K has shown promise as a novel cancer therapeutic agent in a number of studies (20–22), but its utility for the treatment of pediatric cancers has not been previously evaluated. Our initial studies showed that JS-K inhibits the proliferation of A673 and TC71 Ewing sarcoma cells in a dose-dependent fashion (Figure 4A). To assess whether GSTM4 is required for this response, we knocked down GSTM4 in A673 and TC71 cells and then treated control- or GSTM4-silenced cells with increasing concentrations of JS-K. We found that limiting JS-K metabolism by silencing GSTM4 decreased sensitivity to the drug (Figure 4B). These results indicate that GSTM4 is required for JS-K-mediated inhibition of cellular proliferation in Ewing sarcoma cells. We next assessed whether JS-K decreases tumor growth in xenograft models. TC71 cells were injected into the flanks of nude mice and, 24 h after injection, JS-K was administered every other day for a 2-week period. We found that treatment with JS-K significantly reduced tumor growth (Figure 4C, ANOVA Test *p* = 0.023) and increased overall survival (Figure 4D, Mantel–Cox Test, *p* = 0.0002), strongly suggesting that this GSTM4 pro-drug has anti-tumorigenic effects that can be exploited for the treatment of Ewing sarcoma.

## Discussion

Glutathione *S*-transferases comprise a family of enzymes involved in cellular detoxification. Increasing evidence suggests that GSTs play an important role in cancer biology. GST expression is increased in tumors compared with normal tissues (23), resulting in resistance to cancer chemotherapeutic agents (24). Expression of a number of GSTs appears to be inversely associated with patient survival (6), suggesting that GSTs are promising targets for therapeutic intervention.

We, and others, have observed that GSTM4 is a consistently up-regulated GST in Ewing sarcoma cell lines and Ewing, but not other, sarcomas from human patients. GSTM4 is transcriptionally controlled by the fusion oncoprotein EWS/FLI through a specific regulatory element unique to the *GSTM4* promoter. This observation places GSTM4 as a key GST specifically expressed in Ewing sarcoma, a finding with potentially useful therapeutic implications for the treatment of this type of tumor. High GSTM4 expression in primary tumors positively correlates with poor outcomes and increased resistance to etoposide and fenretinide in Ewing sarcoma. In addition, GSTM4 expression is required for oncogenic transformation. Here, we evaluated the potential utility of pharmacologically targeting GSTM4 in Ewing sarcoma. Since GSTM4-specific substrates and inhibitors have not been identified to date, we initially utilized NBDHEX, a broad GST-targeting agent that binds the μ class of GSTs such as GSTM4 with high affinity (25). NBDHEX has been shown to be cytostatic for Ewing sarcoma cells (19) and has synergistic and additive effects when combined with doxorubicin and vincristine, respectively (6, 19). In our studies, NBDHEX decreased cell viability and inhibited oncogenic transformation, and acted synergistically with etoposide in Ewing sarcoma cells. Considering that etoposide resistance correlates with GSTM4 expression in Ewing sarcoma (11), the observed synergistic effects of NBDHEX and etoposide likely are due to inhibition of GSTM4.

We complemented our NBDHEX inhibition studies with a second strategy that took advantage of high GSTM4 expression in Ewing sarcoma. Pro-drugs are inactive precursors that, when metabolized by target cells, generate biologically active agents that affect intracellular events. We tested a GST-activated pro-drug, JS-K, for its effect on the growth of Ewing sarcoma cells *in vitro* and *in vivo*. JS-K was designed as a novel cancer therapeutic agent that releases nitric oxide (NO) when activated by GSTs; this pro-drug has shown promise for the treatment of multiple malignancies, including breast cancer and leukemias (20, 26). Molecular analyses in cellular models showed that JS-K inhibits the growth of Ewing sarcoma cells in a GSTM4-dependent fashion, and that this feature is recapitulated in *in vivo* settings. This is the first study reporting the potential utility of JS-K as a novel pharmacologic agent for the treatment of a pediatric tumor.

Our study also contributes novel mechanistic insight related to the drug resistance induced by GSTM4 in Ewing sarcoma. First, we observed that GSTM4 inhibits etoposide-induced apoptosis by targeting the JNK signaling axis. Second, we demonstrated that the C-terminus of GSTM4 directly or indirectly interacts with the apoptosis-inducing kinase ASK1, limiting phosphorylation of MKK4 by ASK1. This suggests that agents that disrupt the formation of GSTM4/ASK1 complexes or that reactivate the MAPK apoptotic pathway could potentially overcome drug resistance in Ewing sarcoma. The relationship between signaling through the MAPK pathway and drug sensitivity is further supported by a previous report showing that sensitivity to the apoptotic agent fenretinide is dependent on activation of p38 and JNK MAPKs in Ewing sarcoma cells (27).

**Figure 5:**
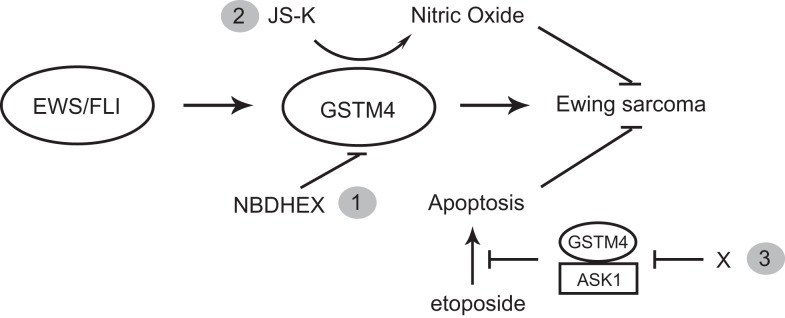
**GSTM4 is a potential therapeutic target for Ewing sarcoma**. GSTM4 is an EWS/FLI-target gene that functionally contributes to the cancer phenotype of Ewing sarcoma by stimulating oncogenic transformation and increasing resistance to chemotherapeutic agents. Modulation of GSTM4 activity through a variety of approaches may offer more effective treatment options for Ewing sarcoma. Inhibition of GSTM4 by inhibitors (e.g., NBDHEX) may decrease transformation and increase the efficacy of chemotherapeutic drugs (1). GSTM4 pro-drugs such as JS-K can be activated in high GSTM4-expressing tumors, leading to localized nitric oxide release and induction of apoptosis (2). Agents that interrupt GSTM4/ASK1 interaction may increase apoptosis induced by chemotherapeutic drugs, facilitating their ability to limit tumor growth (3).

Taken together, our studies point at GSTM4 as a key player and novel therapeutic target in Ewing sarcoma. Targeting of GSTM4 can be achieved via at least three approaches (Figure 5). Firstly, GSTM4 inhibitors such as NBDHEX can be used to limit oncogenic transformation and increase the efficacy of pro-apoptotic chemotherapeutic agents. Secondly, GSTM4-activated pro-drugs such as JS-K can selectively target high GSTM4-expressing malignant cells and spare normal cells and tissues, thus limiting undesirable side effects. Thirdly, specific disruption of GSTM4/ASK1 interactions can potentially reactivate apoptosis, thus decreasing chemoresistance to existing therapeutic agents. Future studies in preclinical and clinical settings are required to fully evaluate the efficacy of targeting GSTM4 as a novel strategy to manage Ewing sarcoma.

## Author Contributions

Wen Luo, Stephen L. Lessnick, Paul J. Shami, Glenn D. Prestwich, and Mitchell S. Cairo contributed to the conception and design of the study. Rupeng Zhuo, Kenneth M. Kosak, Savita Sankar, Elizabeth T. Wiles, Jianxing Zhang, Janet Ayello, and Wen Luo performed experiments. Rupeng Zhuo, Kenneth M. Kosak, Ying Sun, Wen Luo analyzed data. Wen Luo prepared the manuscript.

## Conflict of Interest Statement

Paul J. Shami is a founder, stock holder, and chairman of the board of directors of JSK Therapeutics Inc.
